# Diagnostic Capacity for Invasive Fungal Infections in the Greek Paediatric Haematology-Oncology Units: Report from the Infection Working Group of the Hellenic Society of Paediatric Haematology-Oncology

**DOI:** 10.3390/jof7050357

**Published:** 2021-05-01

**Authors:** Anthi-Marina Markantonatou, Athanasios Tragiannidis, Vasiliki Galani, Dimitrios Doganis, Kondilia Antoniadi, Haroula Tsipou, Maria Lambrou, Nikolaos Katzilakis, Anna Paisiou, Maria Palabougiouki, Marina Servitzoglou, Eugenia Papakonstantinou, Ioulia Peristeri, Efthichia Stiakaki, Eleni Kosmidis, Sophia Polychronopoulou, Antonios Kattamis, Timoleon-Achilleas Vyzantiadis

**Affiliations:** 1 First Department of Microbiology, Medical School, Aristotle University of Thessaloniki, 54124 Thessaloniki, Greece; anthi.marina@gmail.com; 2 Children & Adolescent Haematology-Oncology Unit, Second Department of Paediatrics, Aristotle University of Thessaloniki, AHEPA Hospital, 53646 Thessaloniki, Greece; atragian@auth.gr (A.T.); palabou.m@gmail.com (M.P.); 3 Paediatric and Adolescent Oncology Clinic, Children’s Hospital “MITERA”, 15123 Athens, Greece; vicky_gln@hotmail.com (V.G.); helksom@yahoo.com (E.K.); 4 Oncology Department, “P. and A. Kyriakou” Children’s Hospital, 11527 Athens, Greece; doganisd@gmail.com (D.D.); mservitzoglou@gmail.com (M.S.); 5 Department of Paediatric Haematology-Oncology, “Aghia Sophia” Children’s Hospital, 11527 Athens, Greece; condilia.and@gmail.com (K.A.); sophpol@otenet.gr (S.P.); 6 Paediatric Haematology-Oncology Unit, First Department of Paediatrics, National and Kapodistrian University of Athens, “Aghia Sophia” Children’s Hospital, 11527 Athens, Greece; haroula.tsipou@gmail.com (H.T.); ankatt@med.uoa.gr (A.K.); 7 Department of Paediatric Haematology and Oncology, “Hippokration” Hospital, 54642 Thessaloniki, Greece; mk11106@gmail.com (M.L.); eugepapa@yahoo.gr (E.P.); 8 Paediatric Haematology Oncology Department, University of Crete, 70013 Heraklion, Greece; katzilaher@yahoo.gr (N.K.); efstel@med.uoc.gr (E.S.); 9 Stem Cell Transplant Unit, “Aghia Sophia” Children’s Hospital, 11527 Athens, Greece; salamanca.creta@yahoo.gr (A.P.); iperisteri@yahoo.gr (I.P.)

**Keywords:** invasive fungal infections, audit, diagnostic capacity, paediatric haematolgy-oncology, mycology laboratory, medical mycology

## Abstract

An audit based on a specific questionnaire was attempted, in order to investigate the mycology laboratory diagnostic capacity for invasive fungal diseases (IFDs) in Greek Paediatric Haematology-Oncology departments/units. The study provided the relevant information for the years 2019 and 2020 and included data from all units, concerning culture-based methods and direct microscopy, phenotypic and molecular identification, sensitivity testing, serology and molecular diagnosis, as well as therapeutic drug monitoring. The target was mostly to reveal the level of laboratory coverage for hospitalised paediatric patients, independently of the possibility of performing the tests in the host hospital, or otherwise to refer the specimens elsewhere. In total, the current study demonstrated that the most important facilities and services regarding the IFD diagnostics for paediatric haematology-oncology patients in Greece are available and relatively easily accessible, with a reasonable turnaround time. Acting as an initial registry for further improvements, the audit can serve as a valuable approach to the actual situation and future perspectives. A national clinical mycology network under the auspices of the relevant scientific societies will probably facilitate collaboration between all the departments (clinical and laboratory) involved in invasive fungal infections and provide an easier approach to any necessary test for any hospitalised patient.

## 1. Introduction

Invasive fungal infections (IFIs) comprise an important health issue, especially among immunocompromised patients, such as patients receiving chemotherapy or undergoing haematopoietic stem cell transplantation (HSCT). They are mainly caused by opportunistic pathogens, which can be life-threatening for those with impaired immunity [1,2,3]. As a result, there are factors that increase morbidity and mortality and contribute to long hospitalisation and increased healthcare costs [4,5]. Immunocompromised children are also vulnerable to invasive fungal diseases (IFDs) by opportunistic pathogens [6,7]. Paediatric haematology-oncology patients constitute a considerable part of this population [8,9]. On the one hand, they suffer from a disease that, by nature, impairs their immunity, and on the other hand they receive immunosuppressive treatments that further diminish their ability to properly respond to infection [10]. 

One of the key points in these cases is prevention and timely diagnosis in order to improve patients’ outcome. Best-practice recommendations from international societies and study groups have been published regarding the diagnosis of fungal disease [1,11,12,13,14] as well as proposed standards of care for patients with IFD [13,14,15,16]. In 2014, the European Conference on Infections in Leukemia (ECIL-4) published guidelines for paediatric patients with cancer or allogeneic haemopoietic stem-cell transplantation, concerning the diagnosis, prevention, and treatment of IFIs [7]. Recently, ECIL-8 updated the pre-existing guidelines and recommendations [6], whereas, in 2019, the European Society for Clinical Microbiology and Infectious Diseases (ESCMID) and the European Confederation of Medical Mycology (ECMM) published guidelines regarding the diagnosis of invasive aspergillosis in neonates and children [17]. All the above include guidance about microbiology, histopathology, radiology and clinical investigation. 

In Greece, a study was performed regarding the management of IFDs in adults with haematological malignancies and how this was probably influenced by the financial crisis at the time [18]. The current study intends to investigate the national mycology laboratory diagnostic capacity concerning patients hospitalised in all Greek paediatric haematology-oncology departments/units with the collaboration of the Infection Working Group of the Hellenic Society of Pediatric Hematology-Oncology (IWG HeSPHO), and aims to offer a valuable approach to the actual situation and future perspectives. 

## 2. Methods

The core of the audit was a short questionnaire, created based on previous published experience [11,18] in order to collect information on mycology diagnostic capacity for IFDs. Briefly, it requested information about culture- and microscopy-based methods, non-culture-based methods (serology and molecular), turnaround time for the results, therapeutic drug monitoring (TDM), and the applied treatment strategy (Table 1). 

The questionnaire was distributed during early September 2020 to all (eight) Greek paediatric haematology-oncology departments/units through the network of the Infection Working Group (IWG) of the Hellenic Society of Paediatric Haematology-Oncology (HeSPHO), and was answered by a number of representative clinicians that work in the above units. The units are the Paediatric Haematology-Oncology Unit of the First Department of Paediatrics and the Department of Paediatric Haematology-Oncology of the “Aghia Sophia” Children’s Hospital in Athens, the Oncology Department of the “P. and A. Kyriakou” Children’s Hospital in Athens, the Paediatric and Adolescent Oncology Clinic, Children’s Hospital “MITERA” in Athens, the Childhood and Adolescent Hematology-Oncology Unit of the Second Department of Paediatrics in AHEPA Hospital in Thessaloniki, the Department of Paediatric Haematology and Oncology of the “Hippokration” Hospital in Thessaloniki, the Paediatric Haematology Oncology Department of the General University Hospital of Heraklion in Crete and the Stem Cell Transplant Unit of the “Aghia Sophia” Children’s Hospital in Athens. 

The answered questionnaires were collected in the first weeks of 2021 and provided information for the previous two years, meaning 2019 and 2020.

The main idea was not just to reveal which hospital laboratory was able to perform the specific methodologies or not, but, most importantly and practically, to find out the level of laboratory coverage for the hospitalised paediatric patients, independently of the possibility of performing the tests in the same hospital or referral of the specimens elsewhere (meaning a university or another hospitals’ laboratories, reference laboratories, or even private laboratories). 

## 3. Results

The response rate was 100%, since all eight Greek Departments/Units provided feedback. The units are based in six different hospitals; four general hospitals and two children’s hospitals. Three of the units are university departments. Five of them are located in Athens, two in Thessaloniki and one in the island of Crete. These units’ specialties are Oncology (1), Haematology/Oncology (5), Haematology/Oncology/Infectious Diseases (1) and Bone Marrow Transpantation/Stem Cell Transplantation (2), whereas the most common oncological diseases treated are hematological malignancies (acute leukemias, lymphomas), solid tumors (CNS tumors, neuroblastomas, sarcomas, bone tumors) and myelodysplastic syndromes.

The mean number of hospitalised patients per year, regarding new diagnoses, ranges from 20 to 120 cases, and total hospitalisation number is up to 2,500 per year, depending on the unit. The number of IFDs was remarkably low and mostly concerned infections caused by yeasts and *Aspergillus* spp., as expected. In the last two years, 1-4 invasive candidiases/candidaemias, 1-5 aspergilloses, 0-2 mucormycoses, 0-1 fusarioses, 0-1 infections caused by *Trichosporon* and 0-1 pneumocystoses were reported by each one of the units. (Table 2)

### 3.1. Culture Based Methods 

Regarding identification of the possible fungal isolates, all the supporting microbiology laboratories in the host hospitals have the ability to perform both culture/direct microscopy and phenotypic identification at the genus level and species level, mostly for yeasts and common *Aspergillus* spp., whereas two units are able to perform MALDI-ToF, and one can perform molecular identification as well. The identification is initially performed in the hospital, whereas, in special cases (usually for moulds but also for more rare yeasts), identification is referred to external mycology laboratories with specialised scientists and services. The aforementioned providers may include other hospitals, university/reference laboratories, or even specialised private services. 

The performance of antifungal susceptibility testing (AFST) for both yeasts and moulds is internally available for six out of eight units. In the case of two units, the hospital laboratory performs AFST only for yeasts, whereas mould isolates are referred to university or private laboratories (Table 3).

### 3.2. Non-Culture-Based Methods

The questionnaire included the commonest serology methods, (Immunofluorescence assay (IFA), for *Pneumocystis* as well as PCR, for the most common fungal pathogens. In a few cases, it is possible to perform these tests in the same hospital (Table 4). Usually, they are referred to university/reference, regional or specialised private laboratories. 

In the last two years and depending on the unit, 20-100 *Aspergillus* galactomannan (GM) tests were ordered, as well as 5-80 1,3 β-D-glucan, 0-100 mannan, 0-20 anti-mannan, 0-20 *Aspergillus* antibodies, 0-20 *Cryptococcus* antigen (agglutination and/or lateral flow), 0-5 IFA for *Pneumocystis*, 6-100 PCR tests for *Aspergillus*, 10-100 PCR for *Candida*, 2-5 PCR for *Pneumocystis* and no PCR for *Cryptococcus* (Table 4). 

Turnaround time for the majority of tests ranged from less than 48 hours to 1 week (depending on the test), and is demonstrated in detail in (Table 5).

Regarding the treatment strategy, three out of the eight units applied empirical treatment, while the other four applied pre-emptive, whereas, according to their answers, one unit tried to apply targeted treatment. All the clinicians expressed the opinion that therapeutic drug monitoring (TDM) is very useful; however, it is not a common practice. All the units but two referred to university/reference laboratories or the private sector for TDM (Table 3).

## 4. Discussion

The current survey provides useful information concerning the most important elements for the diagnosis of IFDs in paediatric haematology-oncology patients in Greece. All participating units provided complete and very useful feedback in order to achieve the aim of the study, which was to provide a clear and comprehensive description of the supporting diagnostic capacity in terms of mycology. This fact demonstrates that the clinicians in this field fully realise the importance of good knowledge of the current status in order to take advantage of the available facilities/methodologies and seek improvements when necessary. 

An interesting initial observation is that, according to the answers reported, the IFD rate in children seems to be remarkably lower than that in adults [19]. Probably, this fact is a result of a combination of some important factors. One is children’s hospitalisation conditions, which are carefully arranged and protected. Paediatric haematology-oncology wards are well-maintained regarding the building installations and very strict hygiene rules are applied. Additionally, children’s attendants are almost always their parents, who are extremely careful in following all the necessary precautions in order to protect their children from any kind of infection. Most importantly, it is a known fact that haematology-oncology cases in children are far less frequent than in adults, while co-morbidities are also much fewer, which likely leads to fungal complications being rarer as well.

As was anticipated, the regional-level hospitals that host paediatric haematology-oncology units have adequate laboratory services for the diagnosis of several infections, including mycoses. Additionally, there are well-equipped and experienced university or other institutional laboratories that, by having both a diagnostic and an academic role, contribute greatly to the diagnosis and monitoring of fungal infections. Additionally, there are a few private specialised laboratories that offer relevant services. University medical laboratories, even if situated outside the host hospital, are often closely connected to the hospitals and, in many cases, constitute an entity. Communication and referral is quite easy for the transference of samples and the important bidirectional exchange of information. In all these cases, the vast majority of diagnostics are, in fact, completed “internally” and within the limits of the National Health System, and addressing an external, private facility is only necessary in a few cases. However, even in the previous cases, as in any external referral concerning hospitalised patients, all tests are financially compensated by the social security system.

Regarding culture-based methods, the units seem to have adequate supporting services to identify the majority of the commonest fungal isolates, whereas there is a need for external help in specific cases (mostly for moulds). The laboratories mainly use phenotypical identification procedures, and, more rarely, molecular methods or MALDI-ToF. The latter seems to be available internally in two related laboratories. Generally, molecular identification and MALDI-ToF are referred to external university or private laboratories, as mentioned above. 

Antifungal susceptibility testing is usually performed by the use of commercial (microdilution assays and/or gradient concentration strip methods), and more rarely by in-house-prepared reference microdilution methods. For the majority of the units, this is performed internally in the hospital for both yeasts and moulds and, in some cases, moulds are referred to university or private laboratories. 

Fungal biomarkers are available in only a few cases inside the host hospital, but there is the possibility of performing them externally with a satisfactory turnaround time. This is of great importance, since the use of these biomarkers may contribute to the timely diagnosis and treatment of invasive fungal infections. ECIL-8 suggests performing GM in serum twice a week for children at high risk of invasive fungal disease, whereas GM in BAL and CSF is considered as a significant adjunctive method for the diagnosis of invasive aspergillosis [6]. ESCMID guidelines also support the use of GM as a screening and diagnostic tool [17]. Although the use of 1,3-β-D glucan is not recommended for prospective monitoring or diagnostic use [6,17], it could be a useful approach for children at high risk [12,13,16]. Additionally, the detection of fungal nucleic acids in serum, BAL, other body fluids and tissue specimens is currently recommended for diagnostic use [6]. Paediatric haematology-oncology units in Greece seem to take advantage of the availability of these tests and order them when IFIs are suspected. The most often ordered tests are *Aspergillus* GM, 1,3-β-D-glucan, mannan and PCR for *Candida* and *Aspergillus*.

All the clinicians admitted that TDM is useful for the treatment of their patients, as reported in all current guidelines [6,13,14,16,17]. There is a proven relationship between the concentration of specific antifungal agents and its efficacy, or even toxicity. Thus, if a drug’s absorption, metabolism and/or interactions are difficult to predict, there is a need to apply TDM by measuring its concentration [20]. This ensures that the treatment remains effective and at non-toxic levels. Although the participants realise the importance of applying TDM, in one unit was there the possibility of requesting it internally, and it was only referred in a few cases. It is important to propose a process to facilitate access to relevant laboratories in order to urge clinicians to apply TDM in every case where it is necessary for the proper treatment of their patients.

In conclusion, the current audit demonstrated that the most important facilities and services regarding the invasive fungal disease diagnostics for paediatric haematology-oncology patients in Greece are available and relatively easily to access by the clinicians, with a reasonable turnaround time. Frequent and better communication and interaction between clinical and laboratory doctors is always a key point, which could further contribute to possible improvements. In this context, the formation of a national clinical mycology network under the auspices of the relevant scientific societies will facilitate collaboration between all the departments involved in fungal infections, and probably better organise the mobility and referral of specimens and provide an easier approach to any necessary test for any patient, independently of the unit of hospitalisation. 

## Figures and Tables

**Table 1:** Questionnaire on the diagnostic capacity for invasive fungal infections in the Greek paediatric haematology-oncology units.

**Type of hospital: General Hospital □ University Hospital □ Other □ (please specify)** **Your region:** **Specialties served in your Unit:** **Oncology □ Haematology □ BMT/SCT □ Infectious diseases □ Other □ (please specify)** **Mean number of patients hospitalised per year:** **Please list the 5 most common diseases you have treated during the last 2 years:** **a., b., c., d., e.**			
		**yes**	**no**
Culture based methods:			
**1.**	Ιs there the possibility to perform the following tests in your hospital/institution?		
a.	Cultures for fungal pathogens		
b.	Direct microscopy of the specimen		
c.	Phenotypic identification		
d.	Molecular identification		
e.	MALDI-TOF		
	If any of the answers is no, do you refer the test to another external laboratory (reference laboratory, regional, private, abroad, other)? Please specify.		
**2.**	In case of a positive culture, is there the possibility for identification of the specific pathogens in your institution?		
a.	*Candida* spp.		
b.	*Cryptococcus* spp.		
c.	Other yeasts		
d.	*Aspergillus* spp.		
e.	Mucorales (*Rhizopus* spp., *Mucor* spp., etc)		
f.	*Fusarium* spp.		
g.	*Acremonium /Sarocladium* spp.		
h.	*Scedosporium* spp.		
i.	Other moulds		
	If any of the answers is no, do you refer the test to another external laboratory (reference laboratory, regional, private, abroad, other)? Please specify.		
**3.**	Please specify how the identification is performed in your institution (Phenotypical, Molecular, MALDI-ToF)		
a.	*Candida* spp.		
b.	*Cryptococcus* spp.		
c.	Other yeasts		
d.	*Aspergillus* spp.		
e.	Mucorales (*Rhizopus spp., Mucor* spp.,etc)		
f.	*Fusarium* spp.		
g.	*Acremonium*/*Sarocladium* spp.		
h.	*Scedosporium* spp.		
i.	Other moulds		
	If you refer any of the identification procedures, please specify (which one do you refer and to which type of laboratory-reference, regional, private, abroad, other)		
**4.**	Is there availability of antifungal susceptibility testing in your hospital/institution?		
a.	AFST for yeasts		
b.	AFST for moulds		
	If any of the answers is no, do you refer the test to another external laboratory (reference laboratory, regional, private, abroad, other)? Please specify.		
**5.**	Which of the following invasive fungal infections have you been diagnosed with in the last 2 years? Please provide approximate number.		
a.	Invasive candidiasis		
b.	Aspergillosis		
c.	Mucormycosis		
d.	Fusariosis		
e	Pneumocystosis		
f.	Other (please specify)		
Non-culture based methods:			
**6.**	Is there the possibility to perform the following tests in your hospital/institutional laboratory?		
a.	*Aspergillus* galactomannan		
b.	1, 3-β-D-glucan		
c.	Mannan		
d.	anti-Mannan		
e.	Antibodies *against Aspergillus*		
f.	*Cryptococcus* antigen (agglutination)		
g.	*Cryptococcus* antigen (lateral flow)		
h.	IFA for *Pneumocytis jirovecii*		
i.	PCR for: *Aspergillus* □ *Candida* □ *Cryptococcus* □ *Pneumocystis jirovecii* □		
	If any of the answers is no, do you refer the test to another external laboratory (reference laboratory, region, private, abroad, other)? Please specify.		
**7.**	Which of the following tests have you performed during the last 2 years? Please provide an approximate total number for the tests you have performed. If you referred to an external laboratory, please specify how many “in house” and how many externally.		
a.	*Aspergillus* galactomannan		
b.	1,3-β-D-glucan		
c.	Mannan		
d.	anti-Mannan		
e.	Antibodies *against Aspergillus*		
f.	*Cryptococcus* antigen (agglutination)		
g.	*Cryptococcus* antigen (lateral flow)		
h.	IFA for *Pneumocytis jirovecii*		
i.	PCR for: *Aspergillus* □ *Candida* □ *Cryptococcus* □ *Pneumocystis jirovecii* □		
**8.**	What is the turnaround time for the aforementioned tests?		
a.	*Aspergillus* galactomannan 0–48 h □ 48 h–1 week □>1 week □		
b.	β-D-Glucan 0–48 h □ 48 h–1 week □>1 week □		
c.	Mannan 0–48 h □ 48 h–1 week □>1 week □		
d.	anti-Mannan 0–48 h □ 48 h–1 week □>1 week □		
e.	Antibodies *against Aspergillus* 0–48 h □ 48 h–1 week □>1 week □		
f.	*Cryptococcus* antigen (agglutination) 0–48 h □ 48 h–1 week □>1 week □		
g.	*Cryptococcus* antigen (lateral flow) 0–48 h □ 48 h–1 week □>1 week □		
h.	*Aspergillus* PCR 0–48 h □ 48 h–1 week □>1 week □		
i.	IFA for *Pneumocytis jirovecii* 0–48 h □ 48 h–1 week □>1 week □		
j.	*Candida* PCR 0–48 h □ 48 h–1 week □>1 week □		
k.	*Cryptococcus* PCR 0–48 h □ 48 h–1 week □>1 week □		
l.	*Pneumocystis* PCR 0–48 h □ 48 h–1 week □>1 week □		
**9.**	Is there the possibility to perform TDM for the following antifungal agents? “In house” or externally? Please specify.		
a.	voriconazole		
b.	posaconazole		
c.	isavuconazole		
d.	itraconazole		
e.	5-FC		
**10.**	Do you believe that TDM is useful for the treatment of your patients? Please comment.		
**11.**	What treatment strategy do you usually apply?		
	Empirical □ Pre-emptive □ Targeted □		

BMT/SCT: Bone Marrow Transplant/Stem Cell Transplant, MALDI-ToF: Matrix Assisted Laser Desorption Ionization-Time of Flight, AFST: antifungal susceptibility testing, IFA: Immunofluorescence Assay, TDM: Therapeutic Drug Monitoring.

**Table 2:** Total number of mycoses diagnosed in patients hospitalised in the Greek paediatric haematology-oncology units during the years 2019 and 2020.

Mycoses	Total Number
Candidiases	15–17
Aspergilloses	17–18
Mucormycoses	4
Fusarioses	4
Pneumocystoses	2
Trichosporoses	1

**Table 3:** Capacity of the host hospitals’ laboratories to perform antifungal susceptibility testing (AFST) and therapeutic drug monitoring (TDM) and information about reference to external laboratories.

Unit	Capacity of the Host Hospital’s Laboratory		Refers to An External Laboratory		Type of Laboratory of Reference	
	AFST	TDM *	AFST	TDM *	AFST	TDM *
1	Only yeasts	No	Yes (moulds)	Yes	Private	Private
2	Only yeasts	No	Yes (moulds)	Yes	University	University, Other Hospital, Private
3	Yes	No	No	Yes	-	University, Private
4	Yes	No	No	No	-	-
5	Yes	No	Yes (if necessary)	Yes	University	University
6	Yes	No	No	Yes	-	Private
7	Yes	No	No	Yes	-	University, Private
8	Yes	Yes	No	No	-	-

* The laboratories’ ability for TDM concerns mainly voriconazole, posaconazole and occasionally itraconazole and 5-FC.

**Table 4:** Non-culture-based methods for the diagnosis of IFIs in the patients of the Greek paediatric haematology-oncology units: host hospital’s capacity and total number of tests performed during the years 2019 and 2020.

Diagnostic Tests	Number of Host Hospitals’ Laboratories that Perform the Test	Number of Tests Performed during the Two Years Per Unit
*Aspergillus* galactomannan	2/8	20–100
1,3-β-D-glucan	1/8	5–80
mannan	0/8	0–100
Anti-mannan	0/8	0–20
Antibodies against *Aspergillus*	1/8	0–20
*Cryptococcus* antigen (agglutination)	2/8	0–20
*Cryptococcus* antigen (lateral flow)	2/8	0–10
IFA for *Pneumocystis jirovecii*	1/8	0–5
PCR for:		
*Aspergillus*	2/8	6–100
*Candida*	2/8	10–100
*Cryptococcus*	2/8	0
*Pneumocystis jirovecii*	0/8	2–5

IFA: Immunofluorescence Assay.

**Table 5:** Non-culture-based methods’ turnaround time depending on the unit.

	0–48 h	48 h–1 week	>1 week
*Aspergillus*galactomannan	3/8	4/8	1/8
β-D-glucan	3/8	4/8	1/8
mannan	2/6 *	3/6 *	1/6 *
anti-mannan	2/5 *	2/5 *	1/5 *
antibodies against *Aspergillus*		4/5 *	1/5 *
*Cryptococcus* antigen (agglutination)	2/6 *	4/6 *	
*Cryptococcus* antigen (lateral flow)	2/5 *	3/5 *	
IFA for *Pneumocystis jirovecii*	2/6 *	4/6 *	
*Aspergillus* PCR	2/8	6/8	
*Candida* PCR	2/8	6/8	
*Cryptococcus* PCR	2/6 *	4/6 *	
*Pneumocystis* PCR	1/7 *	6/7 *	

* Answers about the non-culture-based methods’ turnaround time were not available for the total of the Units. IFA: Immunofluorescence Assay.
